# Intestinal Behçet’s disease presenting with intestinal obstruction misdiagnosed as Crohn’s disease: a case report

**DOI:** 10.1186/s13256-025-05749-3

**Published:** 2025-12-12

**Authors:** Jian Song, Liu-Hai Zeng, Wen-Yao Lv, Cheng-Hao Gu, Wei Lin

**Affiliations:** Department of Gastrointestinal Surgery, Xiangshan First People’s Hospital Medical and Health Group, Xiangshan, 315700 Zhejiang Province PR China

**Keywords:** Intestinal Behçet’s disease, Crohn’s disease, Misdiagnosis, Prognosis, Case report

## Abstract

**Background:**

Diagnostic dilemmas persist as intestinal Behçet’s disease mirrors Crohn’s disease with significant overlap in clinical, endoscopic, and pathological features, with up to 30% of patients lacking classic extraintestinal manifestations.

**Case presentation:**

A 45-year-old Chinese male patient was admitted with postprandial abdominal distension and borborygmi for over 1 month, without a history of oral or genital ulcers or other related conditions. Contrast-enhanced abdominal computed tomography demonstrated ileocecal wall thickening with mesenteric lymphadenopathy, while small bowel endoscopy revealed multiple ileal ulcers and relative stenosis of the intestinal lumen. Owing to the tendency for intestinal obstruction, surgical intervention was performed. Pathological findings initially suggested Crohn’s disease. Postoperative anti-inflammatory therapy and nutritional support partially alleviated symptoms, but intermittent abdominal distension persisted. After expert histopathological review by a higher-level institution, the diagnosis was corrected to intestinal Behçet’s disease, with subsequent initiation of thalidomide treatment daily.

**Conclusion:**

Given the strong resemblance between intestinal Behçet’s disease and Crohn’s disease, especially in the absence of typical extraintestinal manifestation, differentiation requires comprehensive endoscopic evaluation and systematic histopathological examination. Although the specific treatment regimen remains controversial, early diagnosis and timely initiation of medical therapy are crucial for improving prognosis.

## Background

Behçet’s disease (BD) is a chronic, relapsing, systemic vasculitic disorder of unknown etiology, primarily characterized by oral ulcers, genital ulcers, skin lesions, and ocular involvement [1]. Less than 5% of BD cases involve the gastrointestinal tract, a condition referred to as intestinal Behçet’s disease (IBD) [2]. The typical endoscopic finding is the presence of large, deep ulcers in the ileocecal region, which may be complicated by perforation, bleeding, or obstruction. Histologically, IBD is characterized by transmural inflammation, making its clinical, endoscopic, and pathological features highly similar to those of Crohn’s disease (CD) [2–4]. Currently, the diagnosis of IBD relies on the International Study Group (ISG) criteria for BD. However, owing to the lack of specific biomarkers and the absence of typical extraintestinal manifestations in approximately 30% of patients, misdiagnosis as inflammatory bowel disease, intestinal tuberculosis, or infectious enteritis is common, leading to delayed treatment [4, 5]. This article reports a patient case initially diagnosed as CD and later reclassified as IBD with the support of a higher-level hospital. By analyzing clinical presentations, endoscopic findings, and pathological features, we aim to explore the key differential diagnostic points between the two conditions and discuss more appropriate treatment strategies.

## Case presentation

**Fig. 1:**
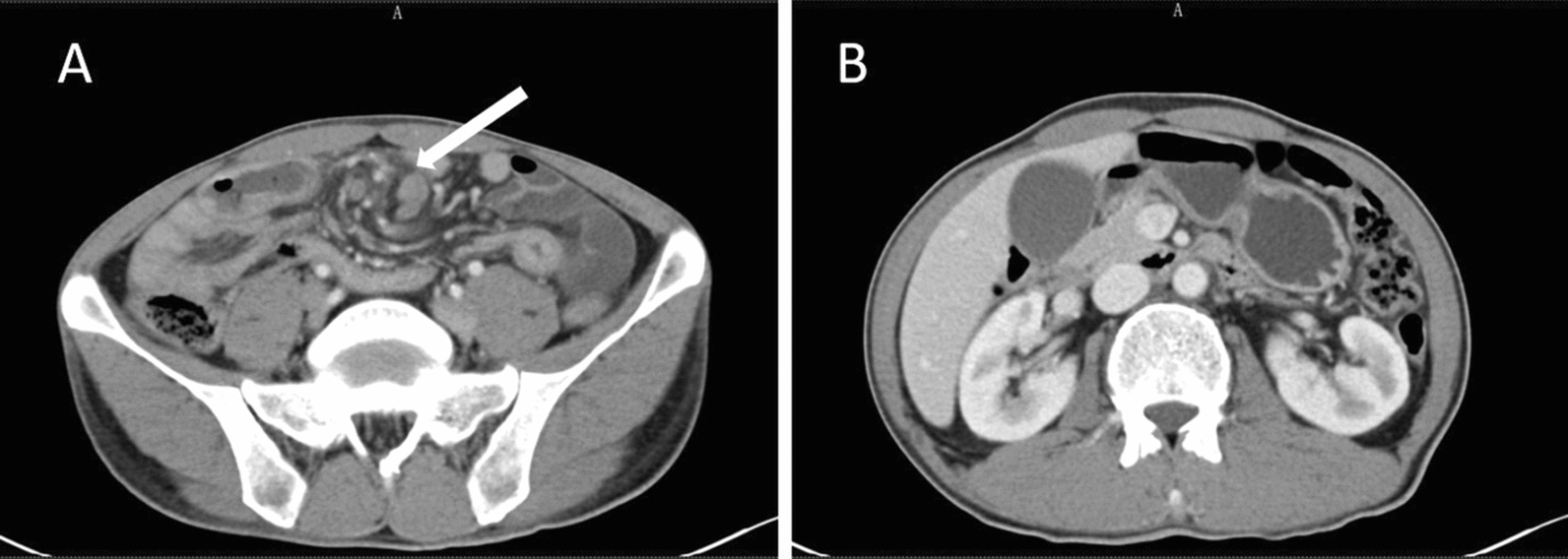
Abdominal enhanced computed tomography: **A** ileocecal wall localized thickening with persistent mucosal enhancement and surrounding mesenteric vessels showing a “whirlpool sign”; **B** progressive dilation of the intestinal lumen suggesting chronic partial bowel obstruction

**Fig. 2:**
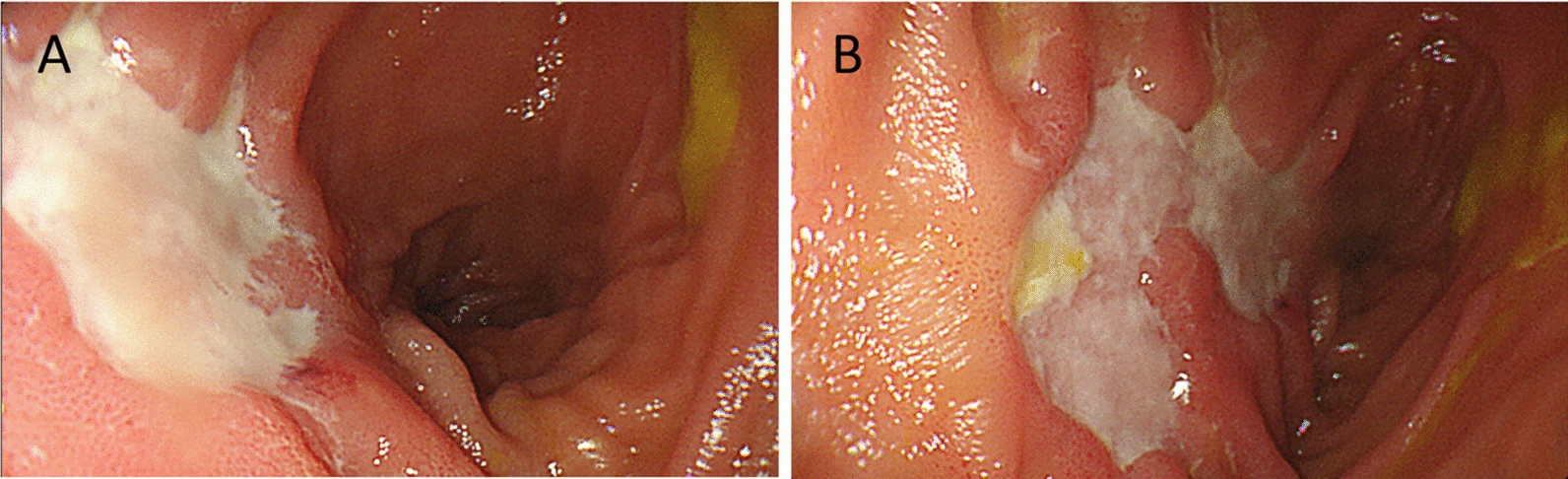
Small bowel endoscopy: **A** multiple round and irregular ulcers in the ileal mucosa; **B** relative narrowing of the intestinal lumen

A 45-year-old Chinese male patient was admitted with postprandial abdominal distension and borborygmi for over 1 month, without a history of oral or genital ulcers or other related conditions. During the course of the disease, he had no fever, bloody stools, or significant weight loss. Previous treatment at an external hospital with antibiotics and antispasmodics was ineffective. Contrast-enhanced abdominal computed tomography (CT) revealed localized thickening of the ileocecal wall (8 mm) with persistent mucosal enhancement, multiple enlarged lymph nodes at the root of the mesentery (short-axis diameter 15 mm), a “whirlpool sign” in the surrounding mesenteric vessels, and proximal small bowel dilation with fluid and fecalith shadows (Fig. 1). Small bowel endoscopy showed multiple round and irregular ulcers in the ileal mucosa with relative intestinal stenosis (Fig. 2). Biopsy pathology indicated a small amount of intestinal mucosal tissue with acute and chronic inflammation, focal necrosis, and granulomatous tissue hyperplasia.

During a multidisciplinary discussion, the radiology department noted that the “whirlpool sign” raised concerns for intestinal volvulus, while confluent lymph nodes and layered intestinal wall enhancement favored CD. The gastroenterology department emphasized ulcer formation with intestinal stenosis, lymphocytic infiltration, and granulomatous tissue hyperplasia, supporting a CD diagnosis despite the absence of typical caseating granulomas. The surgical team comprehensively evaluated the case, highlighting warning signs of intestinal volvulus (whirlpool sign) and progressive proximal small bowel dilation with fecalith formation, suggesting chronic incomplete intestinal obstruction. They concluded that surgical exploration had both diagnostic and therapeutic value.

**Fig. 3:**
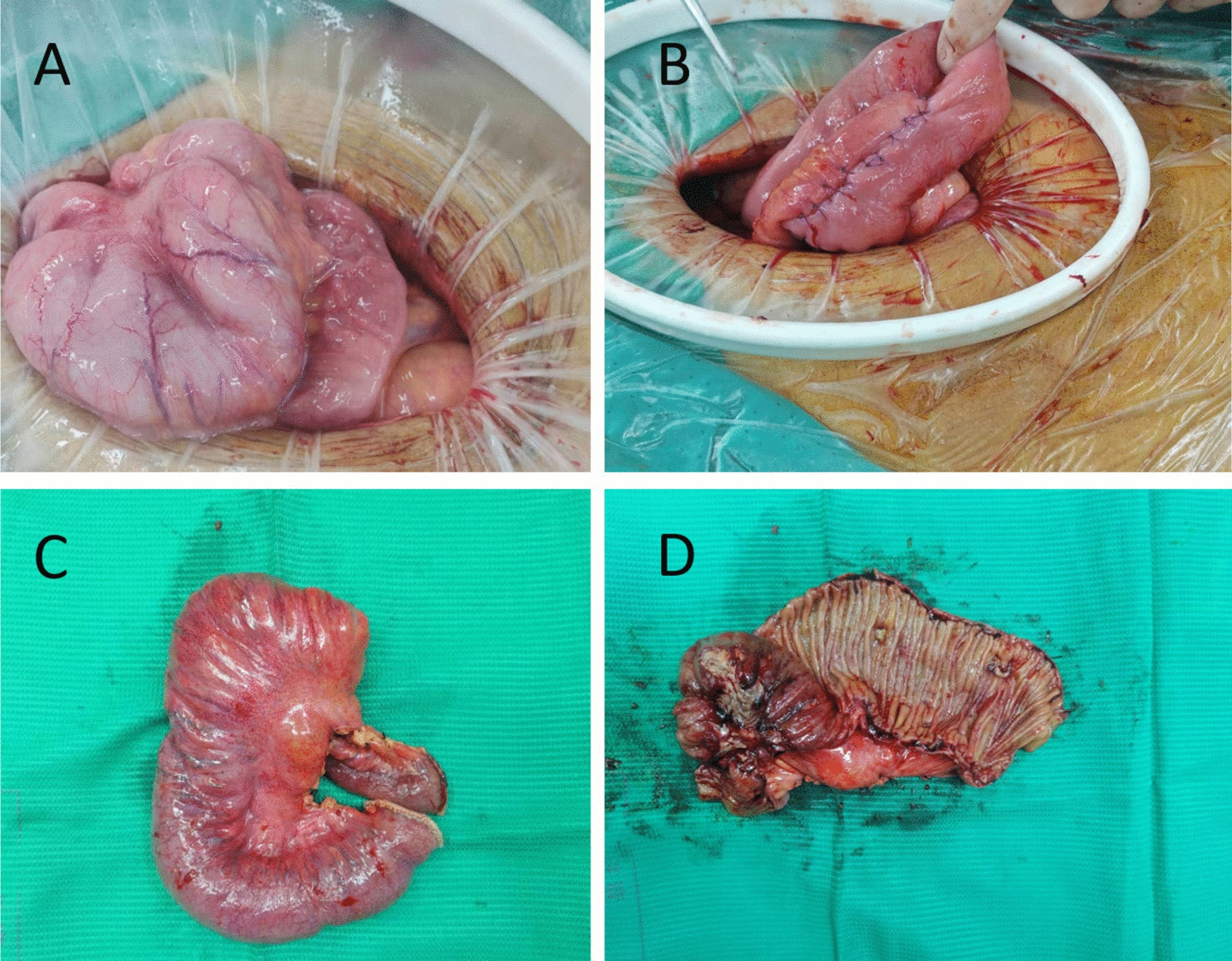
Intraoperative images: **A** ileal segment 20 cm from the ileocecal valve showing adhesions in clusters, with multiple penetrating ulcers on the serosal surface and nodular thickening of the distal 10 cm of the intestinal wall; **B** resection of the diseased ileal segment and side-to-side anastomosis; **C** surgical specimen (unopened); **D** surgical specimen (opened)

**Fig. 4:**
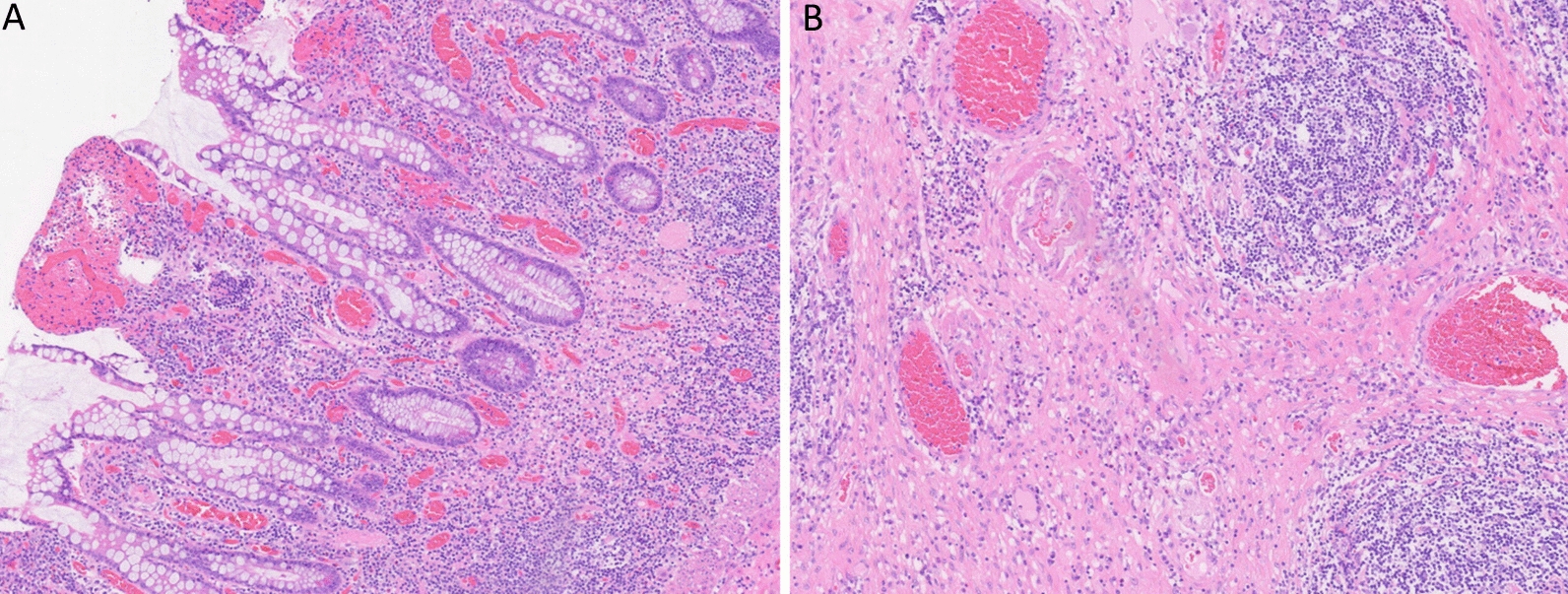
Histopathological findings: **A** multiple ulcers in the intestinal wall with diffuse infiltration of inflammatory cells across all layers and lymph follicle hyperplasia. **B** marked fibrosis and collagen deposition in the serosal layer

Owing to poor conservative treatment outcomes and a tendency toward intestinal obstruction, the patient underwent surgery. Intraoperatively, the ileal segment, which was 20 cm from the ileocecal valve, was found to be adhered in clusters, with multiple penetrating ulcers on the serosal surface and nodular thickening of the distal 10 cm of the intestinal wall. Therefore, it was decided to perform resection of the diseased ileal segment and side-to-side anastomosis (Fig. 3). Postoperative pathology revealed extensive ulceration (some fissure-like), transmural inflammatory cell infiltration with lymphoid follicle hyperplasia, significant serosal fibrosis, and collagen deposition, consistent with CD (Fig. 4).

The patient was diagnosed with CD and received anti-inflammatory and nutritional support postoperatively, with symptom relief. However, 1 month postoperatively, the patient still experienced intermittent abdominal distension. Pathological consultation was requested from Sir Run Run Shaw Hospital, Zhejiang University School of Medicine, which indicated transmural inflammation of the small intestine, full-wall lymph follicle hyperplasia, visible vasculitis, focal vascular wall thickening, and occluded lumens. The diagnosis was revised to IBD. Following the confirmed diagnosis, thalidomide treatment (75 mg/day) was initiated. At the 6-month follow-up, the patient had not experienced any recurrence of abdominal pain, distension, or bowel obstruction.

## Discussion

At the time of the initial diagnosis, the patient lacked typical extraintestinal manifestations and was misdiagnosed with CD after contrast-enhanced abdominal CT, small bowel endoscopy, and histopathological examination of the surgical specimen. This misdiagnosis highlights the significant overlap in clinical, endoscopic, and pathological features between IBD and CD.

Both conditions can present with intestinal symptoms, including abdominal pain, distension, and bloody stools, and endoscopy may reveal intestinal ulcers, mucosal hyperemia, and edema, as well as luminal stenosis, while histopathology may show lymphocyte infiltration, particularly in the mucosal and submucosal layers of the intestine, all of which increase the likelihood of misdiagnosis [6–8]. The critical factor in this case was the absence of the characteristic full-thickness vasculitis seen in IBD in the resected intestinal specimen, which was a major reason for the misdiagnosis. However, key distinguishing features were not fully considered in this case. In terms of endoscopic findings, IBD ulcers are typically more regular, round, or oval in shape, while Crohn’s disease ulcers are irregular, deep, and usually linear or longitudinal, with more irregular edges. The small bowel endoscopy in this patient case had already indicated these distinguishing features, but owing to the limited understanding of IBD at the primary hospital, an effective differential diagnosis could not be made. This lesson emphasizes the importance of solid theoretical knowledge, as it enables the construction of a multidimensional differential system to prevent misdiagnosis in cases of intestinal diseases lacking specific pathological evidence.

Fortunately, with the support of a higher-level hospital, we were able to promptly revise the diagnosis, and the patient received timely supplementation with pharmacological treatment. Overall, there is still no unified international guideline for the treatment of IBD. Although multiple treatment regimens exist, no universally recognized curative medications are available, and there is no consensus on disease remission or standardized guidelines for discontinuing medication [9]. Currently, the main treatment goals for IBD focus on suppressing the inflammatory response, preventing relapse, and delaying disease progression as much as possible [1, 2, 10]. In 2018, the European League Against Rheumatism (EULAR) highlighted that multidisciplinary collaborative care, personalized treatment, and early intervention are crucial strategies for controlling IBD [11]. For patients with severe systemic symptoms or intestinal complications (such as deep ulcers, strictures, fistulas, or bleeding), short-term total parenteral nutrition should be administered, with vigilance for catheter infections and thrombotic risks, and a prompt transition to enteral nutrition. However, in cases of bowel perforation, severe stricture leading to obstruction, large abscesses, or massive gastrointestinal bleeding, surgical intervention is necessary. Unfortunately, patients with IBD face a high risk of postoperative relapse. According to recent research by Park *et al*., the relapse rate within 2 years after surgery is 12.8%, and 20.5% within 4 years, with the risk of relapse increasing as time progresses [12]. Ono *et al*. [13] noted that new lesions after surgery often occur near the anastomosis, possibly due to local immune responses, ischemic injury, and changes in the intestinal microenvironment. Therefore, close monitoring of the anastomotic site after bowel surgery is particularly important.

Current evidence indicates that effective control of perioperative inflammatory activity is the core strategy in reducing the risk of recurrence, with preoperative and early postoperative pharmacological intervention improving postoperative intestinal mucosal healing and significantly lowering the likelihood of recurrence [1, 3, 13]. A study by Choi *et al*. [14] demonstrated that patients who achieved complete remission of intestinal lesions through medical treatment had a lower probability of undergoing surgery compared with those who did not achieve complete remission (13% versus 36% and 43% at 2 and 5 years, respectively; *P* = 0.028). Among patients who did not undergo surgery, the recurrence rates for those who achieved complete remission were 25% at 2 years and 49% at 5 years. Additionally, the extent of intestinal resection was not a key factor influencing postoperative recurrence rates in IBD. Choi *et al*. [14] found that the length of ileocecal resection and whether a hemicolectomy was performed did not significantly affect recurrence or reoperation rates. Limited intestinal resection represents a more appropriate surgical approach, provided that complete lesion clearance or ulcer-free anastomosis is ensured. This further supports the strategy of controlling inflammation with medication before surgery to minimize the surgical extent for patients with IBD. Unfortunately, owing to a lack of experience and technical limitations, this patient was surgically treated without prior drug intervention, which inevitably increases the risk of postoperative recurrence. Moreover, since surgical intervention was performed pre-emptively, it remains unverifiable whether pharmacological management could have improved intestinal function and prevented obstruction. On the basis of prior research findings [1, 3, 13, 14], optimal prognosis may be achieved by judiciously timing surgical intervention after adequate control of inflammatory activity is established.

## Conclusion

For patients with active IBD, we believe that disease activity de-escalation combined with precision-resection strategy should be adopted to minimize surgical trauma while achieving better long-term outcomes.

## Data Availability

The original clinical data, imaging studies, and pathological materials supporting this case report are available from the corresponding author upon reasonable request, subject to institutional review and privacy protection regulations. All patient identifiers have been removed to ensure confidentiality.
